# Early-onset of *ADCK4* glomerulopathy with renal failure: a case report

**DOI:** 10.1186/s12881-017-0392-9

**Published:** 2017-03-16

**Authors:** Ksenija Lolin, Benedetta D. Chiodini, Elise Hennaut, Brigitte Adams, Karin Dahan, Khalid Ismaili

**Affiliations:** 1 Department of Pediatric Nephrology, Hôpital Universitaire des Enfants-Reine Fabiola, Université Libre de Bruxelles (ULB), Avenue JJ Crocq 15, 1020 Brussels, Belgium; 2 Center for Human Genetics, Institut de Pathologie de Gosselies (IPG), Gosselies, Belgium

**Keywords:** Persistent proteinuria, Nephrotic syndrome, Genetics, Case report

## Abstract

**Background:**

We present a rare early presentation of a ADCK4-related glomerulopathy. This case is of interest as potentially treatable if genetic results are timely obtained.

**Case presentation:**

We report the case of a 5-year-old boy who was identified with significant proteinuria by a urinary routine screening program for school children. Physical examination revealed dysplastic ears and abnormal folded pinna. Albumin level was 41 g/L (39–53 g/L), and urine proteins/creatinine ratio was 2.6 g/g. Renal ultrasound showed enlarged kidneys and perimedullary hyperechogenicity. Treatment by angiotensin-converting-enzyme inhibitor was not beneficial. Renal biopsy showed signs of focal segmental glomerulosclerosis. After 4 years of follow-up, he developed a clinical nephrotic syndrome and no response to prednisone and other immunosuppressive agents was obtained. Within 6 months, he was in end-stage-renal-failure (ESRF) and hemodialysis was started. He was transplanted at 10 years with his mother’s kidney. Genes known to be responsible in steroid-resistant nephrotic syndromes were tested. Our patient is compound heterozygous for two mutations in the aarF domain-containing-kinase 4 (*ADCK4*) gene. *ADCK4* gene is one of the genes involved in coenzyme Q10 (CoQ10) biosynthesis, is located in chromosome 19q13.2 and expressed in podocytes. *ADCK4* mutations show a largely renal-limited phenotype. The nephropathy usually presents during adolescence, fast evolves towards ESRF, and may be treatable by CoQ10 supplementation if started early in the disease. Our patient presented nephrotic range proteinuria at 5 years, and he reached ESRF at 10 years.

**Conclusion:**

ADCK4-related glomerulopathy is an important novel and potentially treatable cause of isolated nephropathy not only in adolescents, but also in children in their first decade of life. Discovery of important proteinuria in an asymptomatic child should prompt early genetic investigations.

## Background

Five to 10% of children and adolescents screened at school for proteinuria present a positive urine dipstick (defined as ≥1+). However, only 0.1% of them have persistent proteinuria [1]. This small subset of children might hide a serious renal disease. Isolated persistent proteinuria of nephrotic range (defined as >50 mg/kg/day or urinary proteins/creatinine ratio ≥ 2 g/g) in an otherwise asymptomatic child, is initially investigated by biochemical, viral and immunological analyses. Renal biopsy is also eventually performed. Mutations of genes involved in the podocyte function are traditionally explored mainly in case of steroid-resistant nephrotic syndrome (SRNS), suspicion of syndromic disease or evolution towards renal failure. In the last 20 years, genetics has made tremendous advances in this field. Currently, at least 27 genes are known to cause SRNS [2]. These genetic mutations affect proteins that are expressed in a variety of locations within the podocyte (e.g. the cell membrane, nucleus, cytoskeleton, lysosomes and mitochondria). Altogether, a single-gene cause is found in nearly 30% of cases of SRNS, and probability to identify a single-gene cause is inversely correlated with the age of disease onset [2]. Until few years ago, each gene was investigated separately, and the choice of genes to be firstly screened for mutations depended on the patient’s age, disease presentation and result of renal biopsy [3]. Moreover, patients who did not present in the typical way, risked to be misdiagnosed and unnecessarily treated with toxic immunosuppressive drugs. The evolution of genetics tools and the current use of high-throughput analysis allow nowadays the simultaneous screening of all the known genes implicated in the podocyte development, structure and function and eventually in the development of nephrotic syndrome. The consequences are quicker diagnoses, possibly allowing the avoidance of ineffective treatments and the promptly start of potentially beneficial drugs. Indeed, it seems that about 1% of patients affected with a SRNS carry mutations in genes that function within the coenzyme Q10 (CoQ10) biosynthesis pathway, suggesting that SRNS may be treatable in these individuals with CoQ10 supplementations [2, 4]. Here, we report the case of a patient who showed an unusual clinical presentation of heavy proteinuria and had been only recently diagnosed with the help of the new genetic techniques.

## Case presentation

A 5-year-old boy was identified with proteinuria by a urinary screening program for school children. At that time his medical history was marked by recurrent otitis and tonsillitis, and an episode of bronchopneumonia. Parents were healthy, non-consanguineous and of Caucasian origin. His older brother presented an hypoplasia of the left ear pinna, an atresia of the left external ear canal with a hearing loss. The brother presented no proteinuria, and all the renal investigations resulted as normal. Physical examination of our patient revealed no edema, normal male external genitalia, dysplastic ears with abnormal folded pinna, a short uvula, normal filtrum, a thin superior lip, with normal body weight and height for chronological age. A moderate hearing loss was initially present, but it disappeared after myringotomy, and insertion of ventilation tube was performed.

Blood pressure was normal, no hematuria was observed. Laboratory investigations revealed no hypoalbuminemia (41 g/L), and urine contained nephrotic-range glomerular proteins (proteins/creatinine: 2.6 g/g). All the other biochemical investigations (viral serology and immunological analyses) were normal, and glomerular filtration rate (GFR) measured by creatinine clearance was within the normal range (100 ml/min/1.73 m^2^). Renal ultrasound showed enlarged kidneys and perimedullary hyperechogenicity. A treatment by an angiotensin-converting-enzyme inhibitor was started with no improvement of the proteinuria.

Confronted with this clinical picture of external ear malformations and renal anomalies complicated by heavy proteinuria, a first genetic test was performed few weeks after his first nephrology consultation in order to exclude a branchio-oto-renal (BOR) syndrome. The *NPHS2* podocine gene was also tested, but these first genetic investigations were negative. Renal biopsy showed focal segmental glomerulosclerosis (FSGS) with signs of severe sclerosis in 65% of glomeruli. At age of 8 years, proteinuria increased (proteins/creatinine ratio: 5.4 g/g), renal function deteriorated (plasma creatinine: 0.8 mg/dL), but plasma albumin remained within normal values. At the age of 9 years, he developed a nephrotic syndrome (plasma albumin: 29 g/L) responding neither to a 4 weeks steroid treatment, nor to other immunosuppressive agents (cyclosporine A and mycophenolate mofetil) introduced successively. At the age of 10 years, the child was admitted in the intensive care unit for a 1st and unique non-febrile episode of seizure, in a context of high steroid dosage and hypertension. Five years after his first presentation, he evolved towards an ESRF, and hemodialysis was started. He was eventually transplanted without incidents few months later with his mother’s kidney.

All suspected genes implicated in SRNS have been investigated (NPHS1, CD2AP, PLCE1, ACTN4, TRPC6, INF2, and COQ genes) using a combination of custom designed multi-gene NGS panel for FSGS and related glomerulopathies [5]. High-throughput sequencing was performed on the MiSeq/HiSeq platform (Illumina, San Diego, CA). All findings were confirmed by Sanger sequencing, which was also used to family carrier screening.

**Fig. 1:**
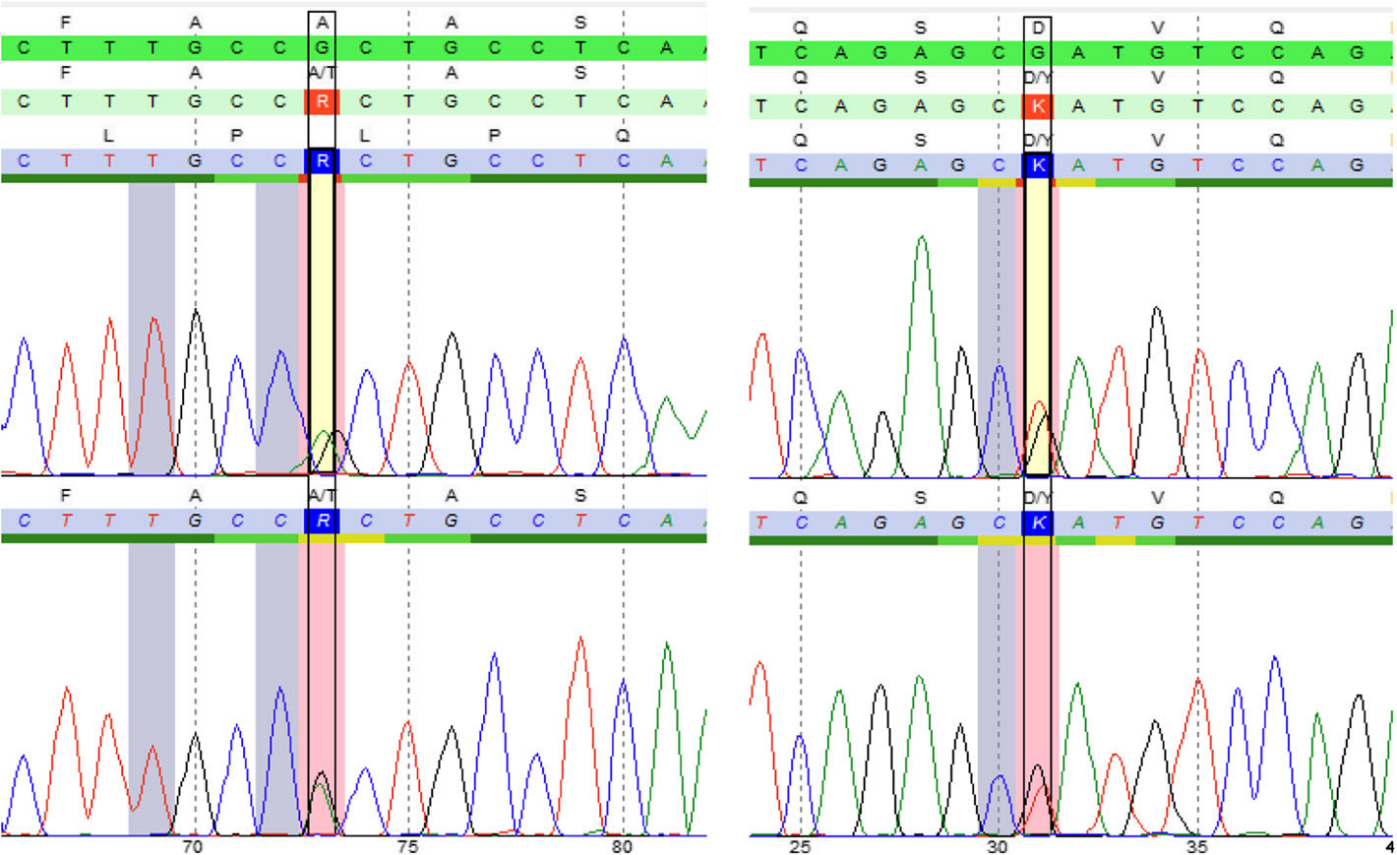
ADCK4 sequence chromatogram showing the two heterozygous mutations found in index patient

**Fig. 2:**
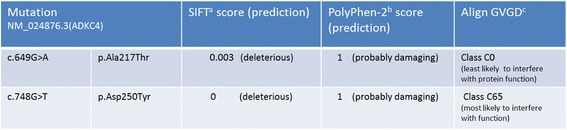
Results of bioinformatics tools applied to predict mutational effects on ADCK4

Mutational analysis revealed a previously reported missense mutation (c.748G > T, residue change p. Asp250Tyr) [5] and a novel sequence variant (c.649G > A, residue change p.Ala217Thr) within the aarF domain containing kinase 4 (ADCK4) gene inherited from both parents (Fig. 1). The c.649G > A change corresponds to a very rare single-nucleotide variant reported in the Exome Aggregation Consortium (ExAC) database with an allele frequency of 0.00015 (18 alleles count/1190492 alleles), mainly observed in the European non-Finnish population (12/18 alleles). It involves a highly conserved residue within the kinase (ABC1 subdomain) and all mutation software’s prediction concluded to a pathogenic effect (https://varsome.com/variant/hg19/adck4%3Ac.649G%3EA, Fig. 2).

## Discussion

Asymptomatic children presenting with heavy proteinuria may eventually develop nephrotic syndrome. Approximately 10% of nephrotic syndrome cases are steroid-resistant, and these patients are at particular risk of developing ESRF. They should be promptly investigated, and genetic causes excluded.

The pathophysiology of SRNS varies, presenting as isolated or as part of a syndrome. As regards to the non-syndromic forms, some are related to a still not completely understood underlying immune disorder, while an increasing number is found to be caused by mutations in genes highly expressed in podocytes (e.g. *NPHS1, NPHS2, CD2AP, PLCE1, ACTN4, TRPC6,* and *INF2*). Syndromic forms of SRNS, may be due to mutations in genes coding transcriptional factors (e.g. *WT1*), glomerular basement membrane components (e.g. *LAMB2*), or mitochondrial proteins as the CoQ10 (e.g. COQ genes) [6].

CoQ10 is a lipid involved in many cellular processes, such as energy production through the mitochondrial respiratory chain, pyrimidine biosynthesis and beta-oxidation of fatty acids. It is an important anti-oxidant, and is also involved in the apoptosis process.

CoQ10 deficiency is implicated in several disorders, such as neurodegenerative diseases, cancer, cardiovascular diseases, diabetes mellitus, and aging. Rarer conditions exist which are caused by a primary or secondary deficiency of CoQ10 in tissues [7, 8].

In humans, to date 18 genes are known to be involved in CoQ10 biosynthesis. Mutations in eight of these genes (*PDSS1*, *PDSS2*, *COQ2, COQ4, COQ6, ADCK3, ADCK4,* and *COQ9*) have been associated with the rare primary forms of CoQ10 deficiency. They are usually transmitted as autosomal recessive traits, and are very heterogeneous clinically and genetically.

These genes have a pleiotropic action, as mutations in the same gene are related to several clinical features. Most clinical phenotypes include numerous combinations of encephalomyopathy, deafness, visual impairment, cerebellar ataxia, seizures and cardiomyopathy. At least four genes include SRNS (*COQ2, COQ6, PDSS2, ADCK4*) [9]. The clinical variability of CoQ10 deficiency concerns not only the pattern of tissue involvement, but also the severity of the disease, the age of onset (from birth to adulthood), and the clinical response to CoQ10 supplementation [7].

In our patient we identified one previously reported missense mutation and a novel sequence variant in the *ADCK4* gene with a pathogenic effect. *ADCK4* is one of the genes involved in CoQ10 biosynthesis. It is located in chromosome 19q13.2, expressed in podocytes and localizes to mitochondria and foot processes [9]. Defects in *ADCK4* are increasingly discovered and up to now 15 different mutations (both frameshift and missense) have been reported in 30 patients affected with SRNS from 17 families without established genotype-phenotype correlations [5, 9]. All these patients presented SRNS and FSGS, predominantly with a largely renal-limited phenotype, and some neurologic dysfunction in 20% of the cases. The disease is mainly discovered in adolescence with a mild-moderate proteinuria with no or mild edema, and a rapid progression towards ESRF (median of 9 months from the diagnosis) [5]. The median age for the need of renal replacement therapy is 16 years [5], which is quite late comparing to patients with other genetic-related nephrotic syndromes. In line with the *ADCK4* cases reported, our patient presented a largely renal-limited phenotype, with no neurological involvement. The isolated dysplastic ear without deafness is probably an unrelated finding. Of interest, the renal disease in our child manifested earlier when compared to the other reported cases [5]: at the age of 5, significant proteinuria resistant to treatment, progressing towards nephrotic syndrome and ESRF 4 and 5 years later, respectively.

Because of the late diagnosis of the genetic defect, we could introduce CoQ10 supplementation only when renal function was already compromised, so that no improvements could have been expected. Conversely, at least a slower progression toward ESRF could have been hoped if the treatment was earlier introduced. A significant decrease of proteinuria was observed in two patients who were started on CoQ10 supplementations while they were still asymptomatic with only a minimal proteinuria and normal kidney function [4, 9].

## Conclusion

Persisting proteinuria even in an asymptomatic child is a worrying sign, which requires extensive investigations. The prompt use of the new-generation genetic tools allowing rapid diagnosis, could avoid the use of potentially toxic treatments. Moreover, if a mutation is found in one of the genes involved in CoQ10 biosynthesis, supplementation with oral CoQ10 may reverse proteinuria and stabilize kidney function if started early in the disease course. *ADCK4*-related glomerulopathy is an important novel and potentially treatable cause of isolated nephropathy not only in adolescents, but also in children in the first decade of life.

## Abbreviations

BOR: Branchio-oto-renal syndrome
CoQ10: Coenzyme Q10
ESRF: End stage renal failure
FSGS: Focal segmental glomerulosclerosis
GFR: Glomerular filtration rate
GVGD: Grantham Variation Grantham Deviation
NGS: Next Generation Sequencing
PolyPhen-2: Polymorphism phenotyping-2
SIFT: Sorting Intolerant From Tolerant
SRNS: Steroid-resistant nephrotic syndrome
